# Managing Recurrent Teratoma in Currarino Syndrome

**DOI:** 10.7759/cureus.48780

**Published:** 2023-11-14

**Authors:** Yat Cheung Chung, Suellyn Centauri, Thang Chien Nguyen

**Affiliations:** 1 Department of General Surgery, Monash Health, Melbourne, AUS; 2 Department of Colorectal Surgery, Monash Health, Melbourne, AUS; 3 Department of Surgery, Monash Health, Melbourne, AUS

**Keywords:** case report, recurrence, teratoma, colorectal surgery, currarino syndrome

## Abstract

To the best of our knowledge, this is the first reported case of a recurrent presacral tumor in Currarino syndrome. Currarino syndrome is a rare disease usually found in childhood with a triad of sacral agenesis, anorectal malformation, and presacral tumor. However, it can often remain undiscovered until adulthood. Currarino syndrome is generally diagnosed during childhood in the setting of recurrent meningitis and is often suspected when there is a family history. Occasionally, it is diagnosed in adulthood through incidental imaging or due to investigations for back pain and chronic constipation. MRI is the recommended imaging modality in this disease process, as it can better help differentiate soft tissue. The tumor can be resected through either the transabdominal approach or the posterior approach (Kraske procedure). We present a 52-year-old female patient who was diagnosed with Currarino syndrome when she was one year old due to recurrent meningitis and surgical resection of a presacral mass and was asymptomatic until she developed back pain and constipation. Her symptoms were investigated with an MRI, revealing a recurrence of a presacral tumor, and she subsequently underwent a Kraske procedure. The patient is currently under annual surveillance, and the residual tumor has remained stable. There are currently no surveillance guidelines after resection of a presacral tumor in Currarino Syndrome. However, follow-up surveillance should be considered.

## Introduction

We present the first reported case of a recurrent presacral tumor in the setting of Currarino syndrome. Although Currarino syndrome is usually diagnosed in childhood, there have been a few case reports of this disease being diagnosed during adulthood. Currarino syndrome often presents with a triad of sacral agenesis, anorectal malformation, and presacral tumor. Currarino syndrome is often identified in adults incidentally on imaging, but MRI is the recommended imaging modality as it helps identify other abnormalities, such as malformations. There are currently two recommended approaches to resecting the tumor: the transabdominal approach and the posterior approach. Given this is the first reported case of a recurrent presacral tumor in Currarino syndrome, there are currently no surveillance guidelines, but we should consider follow-up in symptomatic patients.

## Case presentation

A 52-year-old female patient was referred by her general practitioner to the neurosurgical outpatient clinic for investigation of six months of worsening back pain in the setting of known Currarino syndrome. Her back pain was associated with constipation and bright red blood on wiping post-defecation. She otherwise denied any history of weight loss, anorexia, and abdominal pain. Investigations revealed a recurrent presacral teratoma.

The patient has a past medical history of spina bifida, kidney stones, diabetes, and an open left nephrectomy for chronic pyelonephritis. The patient was first diagnosed with Currarino syndrome after recurrent episodes of meningitis when she was 1 year old. After extensive investigations, she was found to have a presacral mass and underwent resection of a large sacrococcygeal teratoma as a one-year-old.

Currarino syndrome is also evident in her daughter and her granddaughters as it is an autosomal dominant condition with high penetrance in her family.

On examination, the patient had a soft abdomen with a right ileal conduit and a colostomy scar from her initial teratoma excision when she was one year old. Rectal examination showed normal perianal sensation with weak anal tone and minimal squeeze pressure. There was firm stool in the rectum and a palpable soft mass toward the left of the midline.

**Figure 1:**
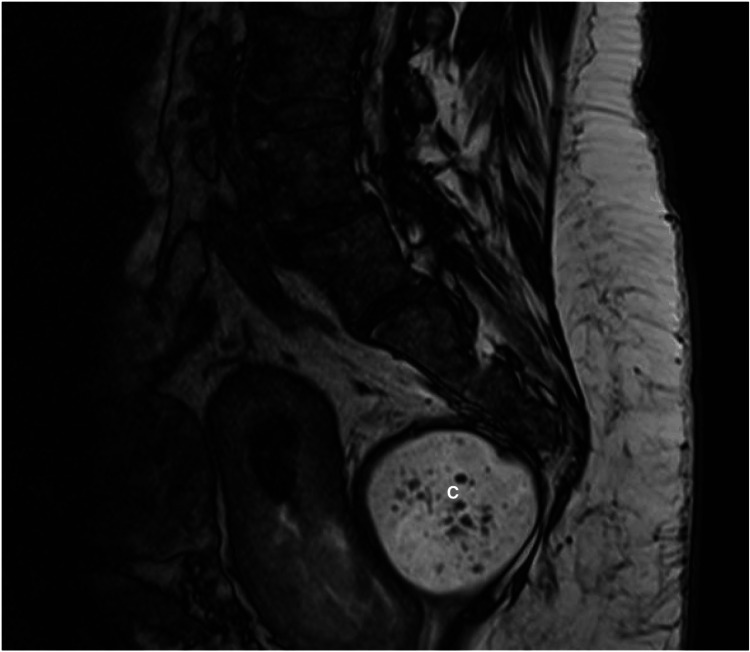
Preoperative magnetic resonance imaging of recurrent teratoma measuring 61 mm × 48 mm × 57 mm (C) (T2, Sagittal Slice 2019).

**Figure 2:**
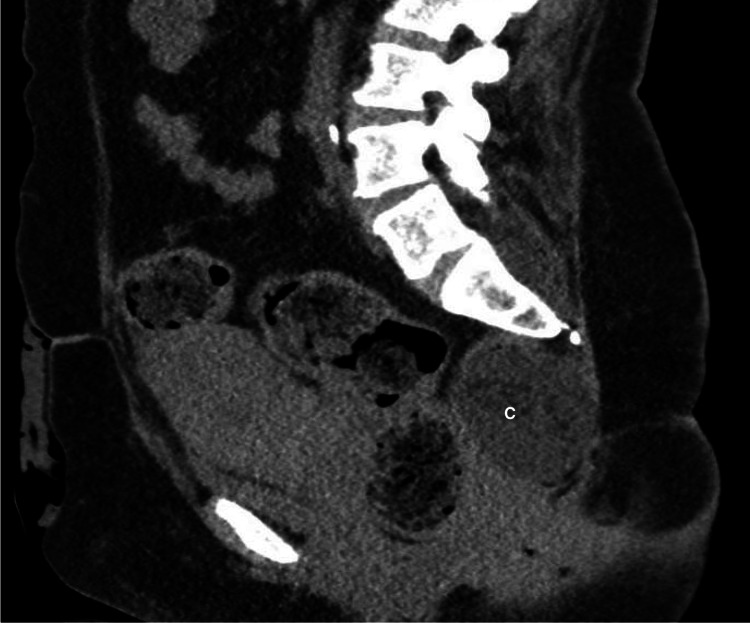
Noncontrast computed tomography intravenous pyelogram sagittal 2016. Teratoma measuring 54 mm × 49 mm × 46 mm (C).

Magnetic resonance imaging (MRI) (Figure 1) demonstrated a 61 mm × 48 mm × 57 mm presacral mass abutting the distal rectum without evidence of invasion of levator ani muscles. It showed innumerable T2 hypointense non-enhancing nodules measuring up to 4mm suggestive of keratin pearls, and T1 hyperintense locules. The tumor was visible on a CT intravenous pyelogram performed three years prior (Figure 2) as a workup before her left nephrectomy but had increased in size in the interim, measuring 54 mm × 49 mm × 46 mm. Given a history of resection of a sacrococcygeal teratoma, these radiological findings were suggestive of a recurrence of presacral teratoma.

The patient underwent a Kraske procedure performed by a colorectal surgeon. A transverse incision was made over the distal sacrum and dissected down to the location of the lesion. Part of the S5 sacrum was removed to aid with the excision of the lesion. The lesion was excised with some spillage. The area was thoroughly washed, and a leak test was performed to ensure that the rectum was intact. The surgery temporarily relieved her back pain. Histopathology confirmed a mature teratoma. Follow-up MRI 14 months after the operation revealed a 13 mm × 31 mm × 8 mm cystic lesion located left of the midline at the level of S2 that was inseparable from the S2 nerve root. Subsequent flexible sigmoidoscopy showed no mucosal abnormality to the rectosigmoid junction and no other mucosal lesions.

**Figure 3:**
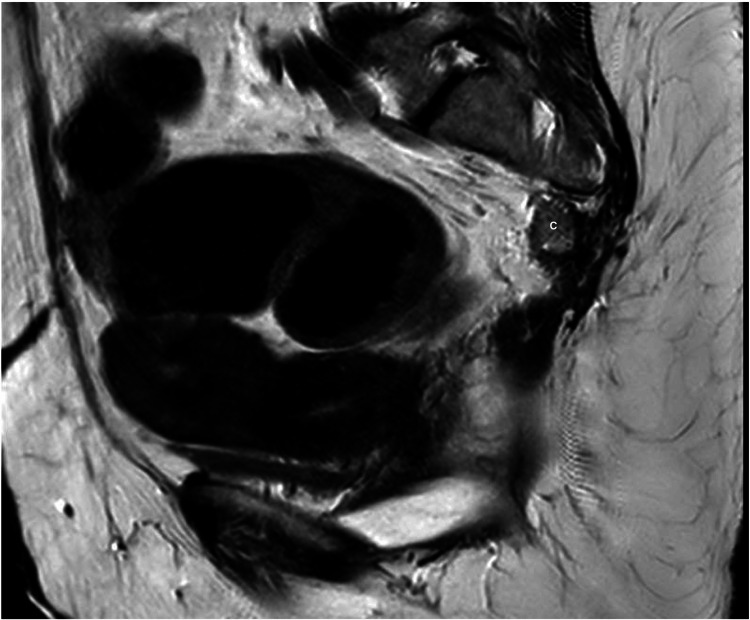
MRI pelvis T2: two-year post-resection of presacral tumor measuring 13 mm × 31 mm × 8 mm (C).

Colorectal and neurosurgical specialist teams concurred that an attempt for further resection would require a laparotomy with the risk of permanent colostomy and possible nerve and major vessel injury. Given the benign nature of the teratoma, surveillance was recommended in the first instance. The cystic lesion has remained stable in size three years after the operation, upon review at the outpatient clinic and on MRI (Figure 3).

## Discussion

Currarino syndrome is a rare (1 in 100,000 births) autosomal dominant genetic condition caused by a mutation in the HLXB9 homeobox gene on chromosome 7. However, many reported sporadic cases exist in the literature. Currarino syndrome is characterized by a triad of (a) sacral agenesis abnormalities, (b) anorectal malformation, and (c) a presacral mass [1]; only one in five cases will have all three abnormalities [2].

The gene exhibits variable phenotypical penetrance. Approximately 80-90% of Currarino syndrome patients have aplasia or hypoplasia of the sacrum and presacral mass, and 5-29% of patients present with abnormal intestine morphology, arteriovenous malformation, or bifid scrotum [1].

Patients are commonly diagnosed in childhood after recurrent urinary tract infections, meningitis, or chronic constipation [3]. Chronic constipation in adults can be multifactorial, categorized into primary colorectal dysfunction, such as slow transit constipation, constipation resulting from other diseases such as Parkinson’s disease, stroke, and opioid use, and chronic idiopathic constipation. However, given the patient's history of Currarino syndrome, a presacral mass should be considered. Occasionally, some cases are detected in adulthood through incidental radiological findings. Identification of a sacrococcygeal bone defect is essential for diagnosing Currarino syndrome. This can vary from mild lateralization of the coccyx to hypoplasia of multiple sacral segments [4]. In our case, there was a bifid sacrum with complete S1 and S2 and rudimentary sacral segments on the right side.

Although there are case reports of Currarino syndrome diagnosed in adulthood, there are currently no reports of recurrent presacral tumors in Currarino syndrome patients.

A variety of anorectal malformations can be present in Currarino syndrome. These can include rectourethral, rectovaginal, and rectovesical fistulas, anal atresia, imperforate anus, and rectal duplication [4]. In our case, MRI and colonoscopy did not identify any fistula.

The presacral space is located between the rectum anteriorly and the sacrococcygeus posteriorly. It is bound superiorly by peritoneal reflections and inferiorly by the levator ani and coccygeus muscles. It usually contains fat, mesenchymal tissue, lymph nodes, nerve plexuses, and blood vessels. Although anterior meningocele and benign teratomas are the most common presacral masses, other masses associated with Currarino syndrome include hamartoma, neuroenteric cysts, or mixed lesions [5]. Other differentials for presacral mass include lipoma, neurofibromatosis, dermoid cyst, enteric cyst, hamartoma, and unclassified tumors [6].

A sacral defect can be identified with a plain x-ray. However, ultrasound, CT, and MRI are helpful in differentiating presacral lesions. MRI is especially useful as it can assess all features of the Currarino triad accurately and help clarify additional pathologies, such as other malformations involving the uterus, vagina, anal sphincter, as well as those involving the spinal cord such as syringomyelia and intradural lipoma [7-8].

There are two main surgical approaches for the excision of a presacral tumor: the posterior approach and the transabdominal approach. We resected the tumor through the posterior approach using a modified Kraske procedure. The posterior approach is usually preferred for tumor diameters of less than 10 cm, as it allows for easier and safer dissection around the coccyx or levator ani muscle. The posterior approach avoids or minimizes entry into the peritoneal cavity, reducing the risk of peritoneal tumor dissemination, intraabdominal adhesion formation, and the risk of future adhesive small bowel obstruction. The disadvantage of the posterior approach is the slightly increased risk of injury to the hypogastric nerves (which provide sympathetic fibers to the pelvic viscera) and the median sacral blood vessels [9].

There are currently no surveillance guidelines for patients with Currarino syndrome. Patients should be counseled that a presacral tumor may recur and that follow-up post-resection is advisable.

## Conclusions

Currarino syndrome presents with a triad of presacral mass, anorectal malformation, and sacral agenesis or hypoplasia. A presacral mass in the context of Currarino syndrome should be investigated with MRI to assist in assessing anatomical variations and to aid in clarifying differential diagnoses. Multidisciplinary discussion is advised to determine the most appropriate surgical approach for managing these variations. Currently, there are no additional case reports of recurrence of presacral tumors in Currarino syndrome, and follow-up in symptomatic patients should be considered to ensure that there is no recurrence.

## References

[1] (2022). National Center for Advancing Translational Sciences, Genetic and Rare Diseases Information Center. U.S. Department of Health and Human Services. https://pharos.ncats.nih.gov/diseases/Currarino%20triad.

[2] Akay S, Battal B, Karaman B, Bozkurt Y (2015). Complete currarino syndrome recognized in adulthood. J Clin Imaging Sci.

[3] Köchling J, Pistor G, Märzhäuser Brands S, Nasir R, Lanksch WR (1996). The Currarino syndrome-hereditary transmitted syndrome of anorectal, sacral and presacral anomalies. Case report and review of the literature. Eur J Pediatr Surg.

[4] Janneck C, Holthusen W (1988). The Currarino triad--a study of 4 cases (Article in German). Z Kinderchir.

[5] Kassir R, Kaczmarek D (2014). A late-recognized Currarino syndrome in an adult revealed by an anal fistula. Int J Surg Case Rep.

[6] Ilhan H, Tokar B, Atasoy MA, Kulali A (2000). Diagnostic steps and staged operative approach in Currarino's triad: a case report and review of the literature. Childs Nerv Syst.

[7] Riebel T, Mäurer J, Teichgräber UK, Bassir C (1999). The spectrum of imaging in Currarino triad. Eur Radiol.

[8] Madhusmita Madhusmita, Ghasi RG, Mittal MK, Bagga D (2018). Anorectal malformations: role of MRI in preoperative evaluation. Indian J Radiol Imaging.

[9] Emoto S, Kaneko M, Murono K (2018). Surgical management for a huge presacral teratoma and a meningocele in an adult with Currarino triad: a case report. Surg Case Rep.

